# Renal Effects of Sulodexide in Type 2 Diabetic Patients without Nephrotic Range Proteinuria

**DOI:** 10.1155/2020/2984680

**Published:** 2020-08-08

**Authors:** Kachonsak Yongwatana, Ouppatham Supasyndh, Bancha Satirapoj

**Affiliations:** Department of Medicine, Phramongkutklao Hospital and College of Medicine, Bangkok, Thailand

## Abstract

**Background:**

Glycosaminoglycan plays an important role in the maintenance of glomerular charge selectivity of diabetic nephropathy. Sulodexide, a mixture of naturally occurring glycosaminoglycan polysaccharide components, has shown a nephroprotective effect in an experimental model of diabetic nephropathy. Although sulodexide reduced albuminuria in patients with type 1 and type 2 diabetes, long-term effects in patients with type 2 diabetes with significant proteinuria have not been established.

**Objectives:**

The study was aimed at investigating the effects of sulodexide on proteinuria and renal function in patients with type 2 diabetes and nephropathy.

**Methods:**

Fifty-two patients with proteinuria between 500 and 3000 mg/day received sulodexide 200 mg/day for 12 months, while 56 matched patients with type 2 diabetes constituted the control group. All patients received standard metabolic and blood pressure controls. Primary outcome was evaluated as percentage of reduced proteinuria compared with the control group. Renal function was assessed using estimated glomerular filtration rate (GFR).

**Results:**

Proteinuria significantly increased in the control group [0.9 (IQR 0.3 to 1.78) to 1.16 (IQR 0.44 to 2.23) g/gCr, *P* = 0.001], whereas it remained stable in the sulodexide group [0.66 (IQR 0.23 to 0.67) to 0.67 (IQR 0.17 to 1.51) g/gCr, *P* = 0.108]. At 12 months, proteinuria was higher by 19.4% (IQR 10.3 to 37.6) in the control group while proteinuria was lower by -17.7% (IQR -53.1 to 3.2) in the sulodexide group with a significant difference between groups (*P* = 0.001). Renal function was noted as a change of estimated GFR, and serum creatinine decreased significantly during the study in both groups but did not significantly differ between groups. No significant changes in the blood pressure, fasting plasma glucose, and hemoglobin A1C were reported.

**Conclusion:**

In addition to standard treatment, sulodexide is efficient in maintaining proteinuria in patients with type 2 diabetes with nonnephrotic range proteinuria, but it did not provide an additional benefit concerning renal disease progression.

## 1. Introduction

Diabetic nephropathy is the main cause of end stage renal disease (ESRD), and the prevalence of nephropathy increased in direct proportion to the prevalence of type 2 diabetes [1, 2]. Novel therapeutic intervention beyond tightening of glycemic control, dietary protein restriction, and strict blood pressure control with angiotensin converting enzyme inhibitors (ACEIs) or angiotensin II receptor blockers (ARBs) should be investigated to reduce the rate of disease progression. Sulodexide, a highly purified mixture of glycosaminoglycans composed of 80% fast moving heparin and 20% dermatan sulfate. In experimental studies, glycosaminoglycans or sulodexide as a heterogenous group of sulfated glycosaminoglycans prevented diabetic renal morphological and functional changes, suppressed renal inflammatory cytokines and vascular growth factors including transforming growth factor-beta (TGF-beta), and improved endothelial dysfunction and albuminuria [3–6]. Initial data suggest the potential value of this treatment to prevent renal disease progression in patients with type 2 diabetes with significant proteinuria.

Proteinuria is known to be an independent risk factor of renal disease progression and ESRDe in the general population and type 2 diabetes [7, 8]. Short-term clinical studies have suggested that glycosaminoglycans improved proteinuria in patients with type 1 and 2 diabetes with albuminuria [9–11], and regarding more clinical data in normoalbuminuric type 2 diabetes, sulodexide treatment prevented the increase of urine TGF-beta [12]. However, renal outcomes especially proteinuria after glycosaminoglycans or sulodexide treatment have not been established in all studies of type 2 diabetic nephropathy [13, 14]. The retrospective study was designed to test the hypothesis that administering sulodexide would decrease proteinuria and renal disease progression in patients with type 2 diabetes without nephrotic range proteinuria compared with treating by standard treatment.

## 2. Methods

This 12-month retrospective cohort study was conducted in patients with type 2 diabetes at the outpatient clinic, Phramongkutklao Hospital. The study was approved by the Institutional Review Board of Phramongkutklao Hospital. Recruitment began in August 2010 and was completed in January 2015. Inclusion criteria comprised age, 18 years or older, and patients with type 2 diabetes with proteinuria between 500 and 3000 mg/day or nonnephrotic range proteinuria. Only patients with proven regular follow-up based on medical reports and prescriptions recorded in our hospital electronic database for a period of 12 months before the study were recruited for the screening visit. Exclusion criteria included type 1 diabetes; pregnancy; active malignancy; severe heart, lung, or liver disease; stroke; chronic infection within one year of starting the study; and any immunological disorders.

All patients with type 2 diabetes on standard care were followed in nephrology clinics every three months. According to the medical history records, eligible patients were divided into two groups based on their regimen 12 months before the study as described below. The treatment group was supplemented with sulodexide 200 mg/day (*N* = 52) and the control group without sulodexide treatment (*N* = 56). The prescriptions were made by the nephrologists monitoring the patients in the period intending to maintain renal function. The main outcome was to evaluate differences in urine protein and GFR rates between the treatment and control groups.

Medical histories, physical examinations, and all laboratory analytes were measured at the beginning and end of the study. All routine laboratory tests including assays for fasting plasma levels of glucose, HbA1c, urea nitrogen, creatinine, and estimated GFR using the 2009 CKD-EPI creatinine equation at baseline and at the end of the study were performed. Adverse events that were or were not considered related to treatment were monitored from medical data sheets.

### 2.1. Statistical Analysis

Measured values of the results were expressed in mean with standard deviation and median with interquartile range (IQR) and percentage. The paired *t*-test was used to compare the change of parameters within groups at baseline and 12 months. Parameters were compared between groups at baseline and 12 months using the chi-square test, Fisher's exact test, Mann-Whitney test, and Student *t*-test. All statistical analyses were performed using SPSS, Version 18.0 for Windows, and statistical significance was set as *P* < 0.05.

## 3. Results

A total of 144 patients were screened for possible study enrollment. One hundred eight patients were eligible according to the entry criteria, and 52 patients received sulodexide treatment. In the sulodexide and control groups, average age was 64.2 ± 10.7 and 66.5 ± 12.8 years, respectively; male prevalence was 61.5 and 66.1%, respectively; and estimated GFR was 49.3 ± 27.5 and 46.3 ± 29.4 mL/min/1.73 m^2^, respectively. All patients received standard treatment with average systolic blood pressure (139.3 ± 16.7 mmHg), diastolic blood pressure (75.5 ± 11.3 mmHg), HbA1c (7.2 ± 1.3%), and prescribed medications including ACEIs/ARBs/ARB (85.4%) and statins (70.2%). Baseline characteristics of the selected patients are reported in Table 1. No significant differences were found in all clinical variables.

**Figure 1:**
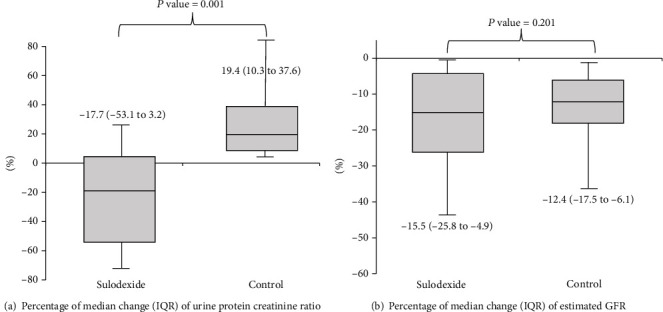
Box-and-whisker-plot diagrams show the (a) percentage of median change of urine protein creatinine ratio and (b) percentage of median change of estimated GFR after 12 months of treatment. (a) shows the percentage of median change that decreased from baseline at -17.7% (IQR -53.1 to 3.2) in the sulodexide group and increased percentage of median change from baseline 19.4% (IQR 10.3 to 37.6) in the control group (*P* = 0.001). (b) shows that estimated GFR levels during the 12-month follow-up significantly declined by -15.5% (IQR -25.8 to -4.9) in the sulodexide group and by -12.4% (IQR -17.5 to -6.1) in the control group, but no significant difference was observed in the rate of change in estimated GFR between the two groups (*P* = 0.201). Error bars represent 95% CIs.

Both sulodexide (49.3 ± 27.5 to 43.5 ± 28.5 mL/min/1.73 m^2^, *P* = 0.001) and control (46.3 ± 29.4 to 41.5 ± 28.6 mL/min/1.73 m^2^, *P* = 0.001) groups exhibited a significant change in estimated GFR levels from baseline during the study (Table 2). GFR levels during the 12-month follow-up significantly declined by -15.5% (IQR -25.8 to -4.9) in the sulodexide group and by -12.4% (IQR -17.5 to -6.1) in the control group (*P* < 0.001), but no significant difference was observed in the rate of change in estimated GFR between the two groups (*P* = 0.201) (Table 3 and Figure 1). Similarly, these results were found in the change of serum creatinine in both groups after 12 months of treatment. The control group showed a significant increase in BUN from 23.8 ± 11.2 mg/dL at baseline to 27.8 ± 13.8 mg/dL at 12 months (*P* = 0.001), but no significant difference in mean change in BUN was observed between the two groups (Table 3).

The control group showed a significant increase in urine protein levels from 0.9 (IQR 0.3 to 1.78) g/gCr at baseline to 1.16 (IQR 0.44 to 2.23) g/gCr at 12 months (*P* = 0.001), but no significant difference was observed in the sulodexide group [0.66 (IQR 0.23 to 0.67) g/gCr at baseline to 0.67 (IQR 0.17 to 1.51) g/gCr at 12 months, *P* = 0.108] (Table 2). However, a significant difference in percentage of change in urine protein was observed between the sulodexide and control groups [-17.7% (IQR -53.1 to 3.2) vs. 19.4% (10.3 to 37.6) g/gCr, *P* = 0.001, respectively] (Table 3 and Figure 1).

No significant variation was observed in the other variables: in particular, the mean values of fasting plasma glucose, HbA1c, and diastolic blood pressure did not change during the study. During the 12-month study, a significant percentage of change in systolic blood pressure [sulodexide: -1.5% (IQR -8.3 to 5.4) vs. control: 4.5% (IQR -1.8 to 10.5), *P* = 0.004] was observed between the sulodexide and control groups (Tables 2 and 3). Sulodexide was well tolerated in this study, and no significant adverse events were reported.

## 4. Discussion

The study was a retrospective clinical trial of sulodexide treatment on renal outcomes in patients with type 2 diabetes. Sulodexide subjects could decrease the percentage of proteinuric progression compared with the control subjects. However, other changes of renal injury parameters including serum creatinine and estimated GFR did not significantly differ in the control group.

The beneficial effects of sulodexide treatment on renal function were limited in our study. The results showed a significant GFR decline over 12 months in patients with diabetes- and nephropathy-prescribed sulodexide therapy, and no significant difference was observed in the rate of GFR decline between the groups. Our results were similar to the results from the randomized, double-blind, placebo-controlled, sulodexide macroalbuminuria (Sun-MACRO) trial. No significant differences were observed in the renal composite end points including doubling of baseline serum creatinine, development of ESRD, or serum creatinine ≥ 6.0 mg/dL between the sulodexide and placebo groups in patients with type 2 diabetes and macroalbuminuria [15]. Currently, sulodexide revealed no selective advantage over standard treatment in preventing GFR progression of diabetic nephropathy in patients with type 2 diabetes.

Overt diabetic nephropathy exhibits progressive albuminuria and renal deterioration over time [16]. Renal outcomes from the clinical studies with sulodexide suggest that proteinuria was reduced when added to a maximal dose of ACEIs or ARBs in patients with type 1 or type 2 diabetes [9–11]. However, the benefit remains inconclusive especially from two multicenter randomized control studies. These two studies demonstrated that sulodexide did not show benefit in reducing albuminuria in patients with type 2 diabetes with normal renal function and microalbuminuria [13, 14]. Our study also indicated that sulodexide treatment tended to lower proteinuria levels while the control group tended to show higher proteinuria levels. Overall, proteinuric outcomes showed that sulodexide was efficient in maintaining proteinuria in patients with type 2 diabetes and nonnephrotic range proteinuria when compared with the control group. The proteinuric outcome was supported by experimental diabetic nephropathy models. Diabetic renal pathological alterations in glomerular basement membrane included partial depletion of anionic glycosaminoglycan and low glomerular anionic charge barrier [17]. Sulodexide or glycosaminoglycans inhibited glomerular basement membrane thickening, reduced glomerular anionic charge, renal signaling pathways, and matrix protein synthesis and albuminuria in diabetic rats and mice [18, 19]. A recent randomized controlled trial demonstrated that sulodexide delayed increasing urine biomarkers in patients with early stage of type 2 diabetes and nephropathy [12]. Finally, meta-analyses supported that sulodexide treatment was associated with a higher proportion of patients that achieved at least a 50% decrease in albumin excretion rate with diabetes and micro- and macroalbuminuria (odds ratio 3.28 (95% CI 1.34-8.06)) [20]. Proteinuric effects of sulodexide were reported in patients with significant albuminuria or proteinuria. Therefore, the positive role of sulodexide treatment might exhibit more evidence of positive outcomes in diabetic nephropathy with significant proteinuria or albuminuria. We did not observe any significant differences in changes of glycemic and blood pressure levels in the sulodexide group. However, there was a significant difference in the changes in systolic blood pressure between the sulodexide group and the control group.

Several limitations were associated with the study. First, the patient selection in our study was not random and could have contained selection bias including compliance to other medications, other nephroprotective therapies, and sodium restriction. However, factors affecting renal disease progression were included and analyzed at baseline of the study, and the confounding factors were similar. Secondly, the present study included a relatively small number of patients and short follow-up time. The relatively short follow-up time may explain the nonsignificant differences in main renal outcomes including renal replacement therapy and end stage renal disease. Thirdly, urine protein and renal function were assessed using urine protein creatinine ratio and estimated GFR equation in our study. They were less accurate than standard 24-hour urine protein collection and radioisotope renal clearance. Finally, as a retrospective study using medical electronic databases, we were unable to confirm whether patients actually took the dispensed medications.

In conclusion, the study indicated that sulodexide treatment prevents rising proteinuria levels in type 2 diabetic nephropathy with significant proteinuria but provided no effect on preservation of renal function. Therefore, long-term clinical trials on a larger scale are warranted to elucidate the benefits that sulodexide affords renal protection.

## Figures and Tables

**Table 1:** Characteristics of the study population.

Variables	Sulodexide group (*n* = 52)	Control group (*n* = 56)	*P* value
Age (yrs)	64.2 ± 10.7	66.5 ± 12.8	0.330
Male (%)	32 (61.5%)	37 (66.1%)	0.329
Body weight (kg)	62.1 ± 10.7	65.1 ± 14.6	0.336
Systolic blood pressure (mmHg)	141.3 ± 18.8	136.3 ± 15.7	0.136
Diastolic blood pressure (mmHg)	76.5 ± 12.0	73.9 ± 12.2	0.289
Comorbid disease (%)			
Hypertension	48 (92.3%)	50 (89.2%)	0.246
Dyslipidemia	48 (92.3%)	51 (91.1%)	0.314
Cerebrovascular disease	7 (13.4%)	5 (8.9%)	0.165
Coronary heart disease	12 (23.1%)	16 (28.6%)	0.426
BUN (mg/dL)	31.9 ± 5.9	23.8 ± 11.2	0.314
Serum creatinine (mg/dL)	1.7 ± 0.8	1.8 ± 0.9	0.658
Glomerular filtration rate (mL/min/1.73 m^2^)	49.3 ± 27.5	46.3 ± 29.4	0.597
Urine protein creatinine ratio (g/gCr)	0.66 (0.23, 0.67)	0.90 (0.3, 1.78)	0.857
Fasting plasma glucose (mg/dL)	135.3 ± 44.9	133.3 ± 39.0	0.804
Hemoglobin A1C (%)	7.3 ± 1.5	7.2 ± 1.2	0.703
ACEIs/ARBs use (*N*, %)	43 (82.7%)	49 (87.5%)	0.418
Statins (*N*, %)	39 (75.0%)	38 (67.8%)	0.121

Data presents as mean with SD, median with interquartile range (IQR) and percentage. ACEI: angiotensin converting enzyme inhibitor; ARB: angiotensin receptor blocker; BUN: blood urea nitrogen.

**Table 2:** Changes of renal outcomes and metabolic parameters after 12 months.

Parameters	Sulodexide group (*n* = 52)			Control group (*n* = 56)		
	Baseline	12 months	*P* value	Baseline	12 months	*P* value
BUN (mg/dL)	31.9 ± 5.9	26.4 ± 14.4	0.481	23.8 ± 11.2	27.8 ± 13.8	0.001
Creatinine (mg/dL)	1.7 ± 0.8	2.1 ± 1.3	0.001	1.8 ± 0.9	2.0 ± 1.1	0.001
GFR (mL/min/1.73 m^2^)	49.3 ± 27.5	43.5 ± 28.5	0.001	46.3 ± 29.4	41.5 ± 28.6	0.001
Urine protein creatinine ratio (g/gCr)	0.66 (0.23, 0.67)	0.67 (0.17, 1.51)	0.108	0.9 (0.3, 1.78)	1.16 (0.44, 2.23)	0.001
Systolic blood pressure (mmHg)	141.3 ± 18.8	135.1 + 23.2	0.078	136.3 ± 15.7	141.8 ± 17.9	0.006
Diastolic blood pressure (mmHg)	76.5 ± 12.0	75.7 ± 15.8	0.679	73.9 ± 12.2	76.2 ± 12.0	0.132
Fasting plasma glucose (mg/dL)	135.3 ± 44.9	130.3 ± 47.1	0.452	133.3 ± 39.0	134.5 ± 36.9	0.829
Hemoglobin A1C (%)	7.1 ± 1.5	7.0 ± 1.1	0.131	7.2 ± 1.2	7.3 ± 1.2	0.283

Data presents as mean with SD and median with interquartile range (IQR). BUN: blood urea nitrogen; GFR: glomerular filtration rate.

**Table 3:** Percentage of changes during 12 months in renal and metabolic outcomes between groups.

Percentage of changes with interquartile range	Sulodexide group (*n* = 52)	Control group (*n* = 56)	*P* value between groups
BUN (mg/dL)	6.0 (-11.6 to 26.6)	12.7 (-4.0 to 33.9)	0.316
Creatinine (mg/dL)	15.8 (5.3 to 27.3)	12.3 (4.2 to 18.8)	0.198
GFR (mL/min/1.73 m^2^)	-15.5 (-25.8 to -4.9)	-12.4 (-17.5 to -6.1)	0.201
Urine protein creatinine ratio (g/gCr)	-17.7 (-53.1 to 3.2)	19.4 (10.3 to 37.6)	0.001
Systolic blood pressure (mmHg)	-1.5 (-8.3 to 5.4)	4.5 (-1.8 to 10.5)	0.004
Diastolic blood pressure (mmHg)	0 (-8.2 to 6.8)	4.9 (-3.8 to 11.9)	0.077
Fasting plasma glucose (mg/dL)	0.8 (-13.9 to 10.9)	5.5 (-6.3 to 16.9)	0.241
Hemoglobin A1C (%)	-1.6 (-10.9 to 7.7)	1.5 (-2.6 to 6.8)	0.254

Data presents as percentage with interquartile range. BUN: blood urea nitrogen; GFR: glomerular filtration rate.

## Data Availability

The Excel of individual clinical data used to support the findings of this study is available from the corresponding author upon request.
